# Complete Genome Sequences of Four Streptococcus canis Strains Isolated from Dogs in South Korea

**DOI:** 10.1128/MRA.00818-20

**Published:** 2020-08-13

**Authors:** Jae-Seok Kim, Shoichi Sakaguchi, Yasuto Fukushima, Haruno Yoshida, Takashi Nakano, Takashi Takahashi

**Affiliations:** a Department of Laboratory Medicine, Kangdong Sacred Heart Hospital, Hallym University College of Medicine, Seoul, Republic of Korea; b Department of Microbiology and Infection Control, Osaka Medical College, Takatsuki, Osaka, Japan; c Laboratory of Infectious Diseases, Graduate School of Infection Control Sciences, Ōmura Satoshi Memorial Institute, Kitasato University, Minato, Tokyo, Japan; University of Rochester School of Medicine and Dentistry

## Abstract

This study reports the complete genome sequences of four strains of Streptococcus canis isolated from dogs in South Korea. Their genomes ranged from 2.157 to 2.265 Mbp, with a G+C content of 39.6% to 39.7%. The sequence types, antimicrobial resistance genes, and S. canis M-like protein alleles were characterized.

## ANNOUNCEMENT

Streptococcus canis, first reported in 1986 (1), forms large, gray/white, smooth colonies and displays beta-hemolysis and the carbohydrate G antigen. In healthy dogs, S. canis is a member of the resident microflora of the oropharynx, skin, genitourinary tract, and anus and can cause self-limiting dermatitis (2). In severe cases, it causes diseases, such as arthritis, streptococcal toxic shock syndrome, necrotizing fasciitis, septicemia, and pneumonia (3, 4). Previously, the draft genome sequences of seven S. canis isolates from diseased companion animals were documented in Japan (5). Here, we report the complete genome sequences of four stored S. canis isolates (identified using mass spectrometry) provided by NosVet in Gyeonggi Province, South Korea (described in Table 1).

**TABLE 1:** Assembly metrics and annotated features of four Streptococcus canis strains isolated from Korean dogs

Strain	Dog species (source)	No. of reads generated by:		Genome size (bp)	Plasmid size (bp)	No. of contig(s)	Mean coverage (×)	*N*_50_ (bp)	No. of CDSs^*a*^	No. of tRNAs	No. of rRNAs	No. of CRISPRs^*b*^	G+C content (%)	Coding ratio (%)	GenBank accession no.	SRA accession no.
		Illumina sequencing	Nanopore sequencing													
HL_77_1	Yorkshire Terrier (ear)	2,404,836	530,407	2,261,128	4,146	2 (including plasmid)	1,691.1	2,261,128	2,140	57	15	2	39.6	84.7	CP053792, CP053793^*c*^	DRR218414, DRR218418
HL_77_2	Yorkshire Terrier (ear)	3,229,118	414,364	2,152,128	5,489	2 (including plasmid)	1,222.8	2,152,128	2,010	67	18	2	39.7	84.6	CP053790, CP053791^*c*^	DRR218415, DRR218419
HL_98_2	Cocker Spaniel (nasal cavity)	3,045,634	603,302	2,176,257		1	1,808.8	2,176,257	2,057	67	18	3	39.7	84.7	CP053789	DRR218416, DRR218420
HL_100	Labrador Retriever (urine)	2,776,570	190,703	2,178,238		1	170	2,178,238	2,078	67	18	2	39.7	84.6	CP046521	DRR218417, DRR218421

a CDSs, coding DNA sequences.

b CRISPRs, clustered regularly interspaced short palindromic repeats.

c A plasmid complete sequence.

The four S. canis strains were inoculated onto 5% sheep blood agar plates and incubated in 5% CO_2_ at 35°C for 24 h. Single colonies were inoculated in genome extraction cultures. Genomic DNA was extracted using the blood and cell culture DNA midikit (Qiagen). For Illumina sequencing, genomic libraries were prepared using the Nextera DNA Flex library prep kit, and sequencing was performed on the Illumina MiSeq platform with a 2 × 150-bp paired-end protocol. We processed the raw reads using fastp v. 0.20.0 (6), with default settings. For Nanopore sequencing, a DNA library was constructed using the rapid sequencing kit (SQK-RAD004). A MinION flow cell (FLO-MIN106; R9.4) was used for sequencing using the MinION software v. 19.12.5 in the standard 48-h sequencing script. Fast5 reads were base called using this MinION software, and the resulting fastq reads were used for assembly by Unicycler. Numbers of reads are shown in Table 1.

The hybrid assembly for three strains (HL_77_1, HL_77_2, and HL_98_2; with two resulting in a single chromosome and a plasmid) was performed using Unicycler v. 0.4.8 (7), and SeqMan Ngen v. 15 was used for the remaining strain (HL_100). Both Unicycler v. 0.4.8 and SeqMan Ngen v. 15 were run in their default mode. Assembly by Unicycler involved the following process: three types of assemblies (Illumina-only assembly, long read plus contig assembly, and long read-only assembly), including bridges, were generated; the quality scores were assigned to each bridge; and the most supportive bridge was selected. After this assembly, Unicycler carried out multiple rounds of polishing with Racon to improve the sequence accuracy. The chromosome and plasmid sequences were annotated using the Prokaryotic Genome Annotation Pipeline (8). Assembly metrics (genome sizes, numbers of contigs, mean coverages, and *N*_50_ values) and annotated features (numbers of coding DNA sequences/tRNAs/rRNAs/clustered regularly interspaced short palindromic repeats, G+C contents, and coding ratios) are shown in Table 1.

**FIG 1:**
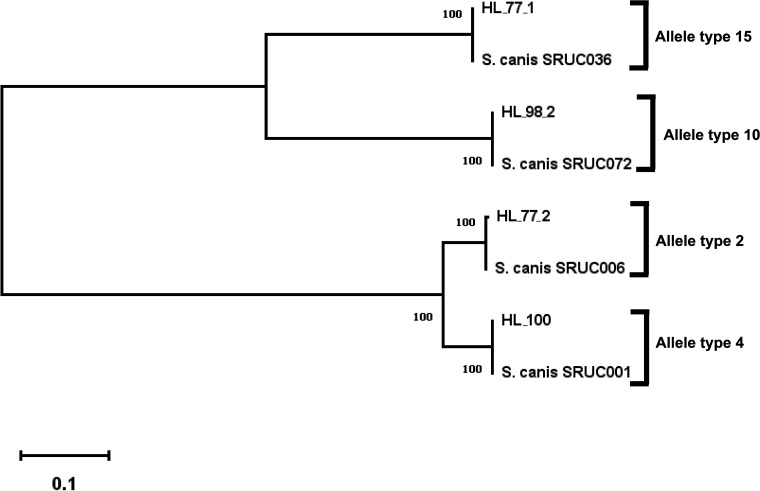
Phylogenetic tree of deduced S. canis M-like protein (SCM) in strains HL_77_1, HL_77_2, HL_98_2, and HL_100 by the neighbor-joining method. The SCM in S. canis strains SRUC001 (GenBank accession number MH996657.1), SRUC006 (MH996659.1), SRUC036 (MH996667.1), and SRUC072 (MH996676.1) were applied as internal controls.

We determined the sequence types (STs) (allelic profile: *gki*, *gtr*, *murI*, *mutS*, *recP*, *xpt*, and *yqiZ*) and antimicrobial resistance (AMR) genotypes by analyzing our contig sequences on the Web-based applications MLST v. 2.0 (https://cge.cbs.dtu.dk/services/MLST/) and ResFinder v. 3.2 (https://cge.cbs.dtu.dk/services/ResFinder/), which are managed by the Center for Genomic Epidemiology (9, 10). Strain HL_77_1 belongs to ST1, strain HL_77_2 to ST2, strain HL_98_2 to ST15, and strain HL_100 to ST13. Furthermore, we found the AMR genes *tet*(O), *tet*(M), *tet*(S), and *tet*(M) in strains HL_77_1, HL_77_2, HL_98_2, and HL_100, respectively. The nucleotide sequences encoding S. canis M-like protein (SCM) were extracted from the genomic data, and types of the SCM allele were deduced based on their amino acid sequence variations. Phylogenetic analysis was performed as previously described (11–13). We observed two groups in the phylogenetic tree, namely, one containing HL_77_2 and HL_100, which constituted SCM alleles 2 and 4, and the other group, which consisted of SCM alleles 10 (HL_98_2) and 15 (HL_77_1) (Fig. 1).

### Data availability.

The complete genome sequences of these four strains have been deposited in DDBJ/EMBL/GenBank under accession numbers CP053792, CP053793, CP053790, CP053791, CP053789, and CP046521 and SRA accession numbers DRR218414, DRR218415, DRR218416, DRR218417, DRR218418, DRR218419, DRR218420, and DRR218421.
